# Remodeling of Tumor Immune Microenvironment by Oncolytic Viruses

**DOI:** 10.3389/fonc.2020.561372

**Published:** 2021-02-19

**Authors:** Bin Zhang, Xilei Wang, Ping Cheng

**Affiliations:** State Key Laboratory of Biotherapy and Cancer Center/Collaborative Innovation Center for Biotherapy, West China Hospital, Sichuan University, Chengdu, China

**Keywords:** oncolytic virus, tumor immune microenvironment, immune cells, cytokines, immune checkpoints

## Abstract

Oncolytic viruses (OVs) are potential antitumor agents with unique therapeutic mechanisms. They possess the ability of direct oncolysis and the induction of antitumor immunity. OV can be genetically engineered to potentiate antitumor efficacy by remodeling the tumor immune microenvironment. The present mini review mainly describes the effect of OVs on remodeling of the tumor immune microenvironment and explores the mechanism of regulation of the host immune system and the promotion of the immune cells to destroy carcinoma cells by OVs. Furthermore, this article focuses on the utilization of OVs as vectors for the delivery of immunomodulatory cytokines or antibodies.

## Introduction

Oncolytic viruses (OVs) are naturally occurring or genetically engineered viruses that can selectively target malignant tissues while reducing the infection intensity in the normal tissues (1). As a potential treatment modality against cancer, the past two decades has witnessed a breakthrough in oncolytic virotherapy. The Food Drug Administration (FDA) approved the first oncolytic agent T-VEC for the treatment of patients with melanoma in 2015. T-VEC is an attenuated herpes simplex virus type 1 (HSV-1) that was genetically modified to encode the granulocyte-macrophage colony-stimulating factor (GM-CSF) (2). Unlike in the general gene therapy, OVs not only serve as vectors for transgenic delivery but also as active pharmaceutical agents.

The anti-tumor activity of OVs involves a variety of mechanisms, including the natural interactions among tumor cells, viruses, and the immune system (3). Although it has not been fully confirmed, the mechanism underlying the anti-tumor activity of OVs can mainly be categorized into two types. One is the selective killing of tumor cells by OVs. However, this effect is influenced by the expression of cell surface receptors and the antiviral response of the host cells. The other mechanism of OV-mediated anti-tumor activity is associated with the induction of systemic anti-tumor immunity. The local delivery of immune regulatory factors by viral vectors is beneficial in creating create a proinflammatory tumor microenvironment, which in turn promotes the systemic antitumor immunity (4). Furthermore, the release of tumor-associated antigens (TAAs) after tumor cell lysis can promote the adaptive immune responses, which in turn mediates tumor regression at distant tumor sites that are unexposed to the virus (4). Presently, a variety of OVs have been developed for the treatment of cancer in pre-clinical and clinical trials, including the Newcastle disease virus (NDV) (5), reovirus (6), adenovirus (7), and vesicular stomatitis virus (VSV) (8), among others. **Table 1** lists the several OVs that are currently underway for clinical trials and their indications.

**Table 1:** List of part of ongoing clinical trials of OVs on Clinical trials.gov.

Viral backbone	Oncolytic virus	Gene modification	Combination	Indication	Status/phase	ClinicalTrial.gov identifier
Adenovirus	DNX-2401	Δ24-RGD insertion	/	Glioblastoma	Recruiting; phase 1	NCT03896568
Adenovirus	ORCA-010	Δ24-RGD insertion; insertion of T1 mutation in E3/19K gene	/	Prostate cancer	Recruiting; phase 1/2a	NCT04097002
Adenovirus	LOAd703	Encoding for TMZ-CD40L and 4-1BBL	Gemcitabine+nabpaclitaxel+/-atezolizumab	Pancreatic cancer	Recruiting; phase 1/2a	NCT02705196
Vesicular stomatitis virus	VSV-IFNβ-NIS	Encoding for interferon β (IFNβ) and the sodium iodide symporter (NIS)	Pembrolizumab	Non-small cell lung cancer; head and neck squamous cell carcinoma	Recruiting; phase 1	NCT03647163
Vesicular stomatitis virus	VSV-IFNβ-NIS	Encoding for interferon β (IFNβ) and the sodium iodide symporter (NIS)	Cemiplimab	Melanoma; hepatocellular carcinoma; non small cell lung cancer; endometrial cancer	Recruiting; phase 2	NCT04291105
Herpes simplex virus	G207	Deletion of both γ134.5 loci; insertional inactivation of UL39	/	Cerebellar brain tumor	Recruiting; phase 1	NCT03911388
Herpes simplex virus	OH2	Encoding for human granulocyte macrophage colony-stimulating factor (GM-CSF)	/	Pancreatic cancer	Recruiting; phase1b/2	NCT04637698
Herpes simplex virus	ONCR-177	Encoding for IL-2, CCL4, FLT3L, antagonists CTLA-4 and PD-1	Pembrolizumab	Solid tumor	Recruiting; phase 1	NCT04348916
Newcastle disease virus	MEDI5395	Encoding for GM-CSF	Durvalumab	Solid tumor	Recruiting; phase 1	NCT03889275
Reovirus	Pelareorep	Wild-type variant	Paclitaxel+avelumab	Metastatic breast cancer	Recruiting; phase 2	NCT04215146
Reovirus	Pelareorep	Wild-type variant	Pembrolizumab	Pancreatic cancer	Active, not recruiting; phase 2	NCT03723915
Measles virus	MV-s-NAP	Expressing the helicobacter pylori neutrophil-activating protein	/	Metastatic breast cancer	Recruiting; phase 1	NCT04521764
Measles virus	MV-NIS	Expressing the sodium-iodide symporter (NIS)	/	Medulloblastoma; atypical teratoid rhabdoid tumor	Recruiting; phase 1	NCT02962167
Poliovirus	PVSRIPO	Containing a heterologous internal ribosomal entry site (IRES) derived from the human rhinovirus type 2	/	Invasive breast cancer	Recruiting; phase 1	NCT03564782
Poliovirus	PVSRIPO	Containing a heterologous internal ribosomal entry site (IRES) derived from the human rhinovirus type 2	/	Recurrent malignant glioma	Recruiting; phase 1b	NCT03043391
Vaccinia virus	T601	Deletion of genes of thymidine kinase and ribonucleotide reductase; insertion of FCU1 gene	Flucytosine	Malignant solid tumors	Recruiting; phase 1/2a	NCT04226066
Vaccinia virus	TBio-6517	Expressing an anti-CTLA-4 antibody, Flt3 and IL-12	Pembrolizumab	Advanced solid tumors	Recruiting; phase 1/2a	NCT04301011
Coxsackievirus	V937	Wild-type variant	Pembrolizumab	Advanced/metastatic solid tumors	Recruiting; phase1b/2	NCT04521621

## A Brief Description of the Tumor Immune Microenvironment (TIME)

Tumor microenvironment is a sophisticated niche of developing cancerous cells and various non-cancerous components; and the latter mainly consists of cancer-associated fibroblasts (CAFs), extracellular matrix (ECM), vascular endothelial cells, tumor-associated immune cells, as well as soluble substances such as cytokines and chemokines (9). These components together constitute an environment that is conducive for the maintenance of tumor cell growth. They are involved in immune modulation in the tumor microenvironment to varying degrees, even for non-immune cells such as CAFs. Past studies have suggested that not only do CAFs induce the epithelial-mesenchymal transition (EMT) by secreting multiple growth factors, they also, exhibit immunosuppressive phenotypes because of the expression of the inhibitory surface proteins such as PD-L1 (10).The analysis of the immune profiles of tumor is helpful to predict the disease progression and to customize the treatment regimen. The TIME may exhibit distinguishing immunological status based on the heterogeneity in the cell populations, diseases and patients. Currently, three different classes of TIME have been proposed, including inflamed, excluded and deserted TIME (11). Inflamed TIMEs, or “hot” tumor, are characterized by the abundant accumulation in the tumor core and the stroma of T cells expressing PD-1 and/or CTLA-4, myeloid cells and monocytes. It is however simultaneously accompanied by several proinflammatory cytokines. Inflamed tumors are often positively associated with patients’ responses to cancer immunotherapy (12). The excluded TIMEs are also quite abundant in the immune cells, albeit there is a relative void of immune effector cells in the tumor core. These immune cells are mainly present at the border of the tumor mass possibly due to the absence of specific chemokines and the presence of substantial barriers or specific inhibitors (13). The deserted TIMEs are considered to be immunological “cold” tumor. The typical indication of this status is the lack of immune cells and cytokines in either the core or in the stroma of the tumor mass.

**Figure 1:**
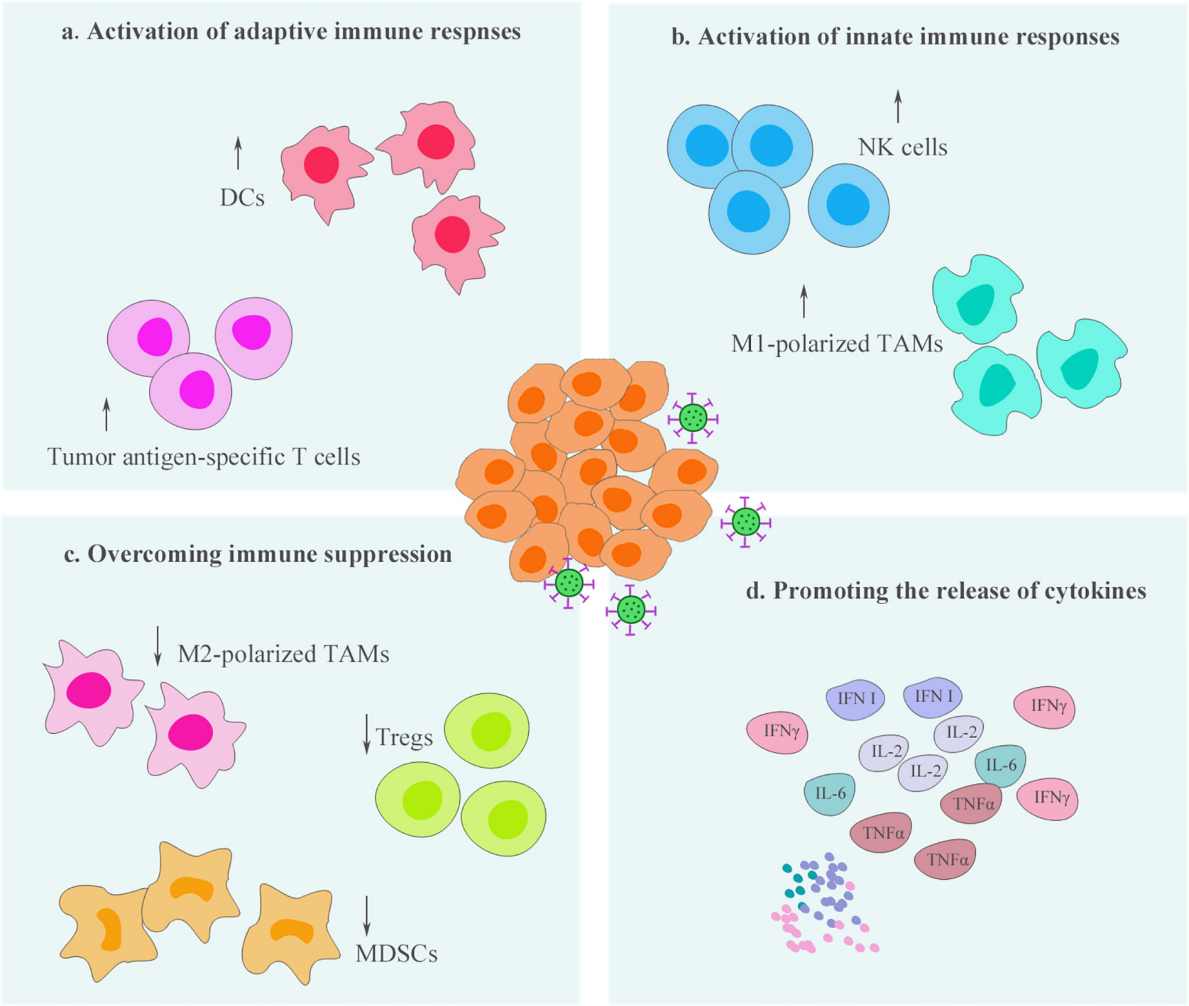
Oncolytic virotherapy process the ability to remodel the tumor immune microenvironment (TIME). Oncolytic viruses (OV) infection can enhance the infiltration and activity of immune cells, including innate and adaptive immune cells, within the TIME **(A–B)**. At the same time, these therapeutic viruses reduce the populations of immunosuppressive cells and promote the immunophenotypes of immune cells shift toward antitumor status, thereby, overcoming immune suppression within the TIME **(C)**. In addition, activation of antitumor immunity by oncolytic virotherapy is often accompanied by the production of a variety of proinflammatory cytokines, which is beneficial to further “hot” the TIME **(D)**.

Effectual oncolytic virotherapy is closely linked to the tumor microenvironment. On one hand, tumor microenvironment may limit the efficacy of OVs. For example, viral transmission after intratumoral injection may be weakened by the substantial barriers present at the tumor mass, such as the dense ECM network (14). The degradation of ECM by the use of relaxin or specific enzymes, such as hyaluronidase, has been demonstrated to promote viral spread among tumor tissues (15, 16). In addition, elevated interstitial hydrostatic pressure caused by fibrosis and vascular abnormalities in tumors have been reported to provide another barrier to oncolytic virotherapy (17). There are a few related reviews that have elaborated on the challenges of tumor microenvironment to OVs and have proposed reasoned solutions (18). On the other hand, OVs serve as powerful immunological stimuli and possess the ability of remodeling of the TIME (**Figure 1**), which is also the focus of this opinion article. OVs influence the entire immunological process *via* multiple mechanisms and promote the recruitment and activation of immune cells. Thus, the capability of OVs to “hot” TIME can enhance the sensitivity of tumors to immunotherapy (19).

## Remodeling the TIME *Via* Oncolytic Virotherapy

### Recruitment and Activation of Dendritic Cells (DCs)

DCs are well-known antigen-presenting cells (APCs) that are responsible for processing and presenting antigens to the effector cells in the context of the major histocompatibility (MHCs). They play an important role in the initiation of innate and adaptive immune responses, particularly, matured DCs provide T cells co-stimulatory signals and are essential for T cell priming (20). However, it is often inadequate to induce potent antitumor immune responses in a tumor mass due to the immunosuppressive TIME. Currently, several researchers have proposed anticancer agents that target of DCs, such as the DC vaccines aiming at activation and accumulation of functional DCs (21). Alternatively, activating DCs *via* oncolytic virotherapy can unleash T cell responses, which is also a potential therapeutic strategy. After OV infection, the innate immune system rapidly recognizes virus particles and promotes DC maturation. Although viral infection initially induces a virus-targeting immune response, DCs process the ability to cross-present tumor antigens to cytotoxic T lymphocytes, thereby initiating a tumor-specific immune response (22).

Oncolysis induced by a recombinant poliovirus-rhinovirus chimera exposed the tumor antigens, while DCs co-cultured with the supernatant from the chimera-infected tumor cells exhibited increased expression of type I IFNs, CD40, CD80, and CD83, suggesting that this virus promotes APC maturation (23). A high proportion of splenic CD11c^+^CD8^+^DCs was detected in a mouse model treated with an engineered adenovirus, moreover, tumor-infiltrating plasmacytoid DCs displayed a mature phenotype with the ability to prime tumor-specific cytotoxic T cell responses (24). In addition, several studies have demonstrated that other OVs, such as vaccinia virus (25), measles virus (26), and HSV (27), can also enhance the antigen presentation ability of DCs, which is often accompanied by an increased expression of costimulatory/activation molecules, such as CD80, CD86, and MHC II. OVs assist DCs in its functions in two main ways. First, OVs promote DCs to recognize the tumor antigens by upregulating antigen presentation pathways in tumors. It is already well-established that, to evade immunosurveillance, tumor cells upregulate immune-inhibitory surface receptors and downregulate functional molecules related to antigen processing and presentation (28, 29). In this setting, OVs can overcome some evasion strategies. For example, an ovarian cancer cell line exhibited higher expression of MHC class I and other molecules involved in antigen processing, such as the transporter associated with antigen processing (TAP) and β2-microglobulin (β2M) when exposed to an oncolytic reovirus. This effect promoted DC maturation, which resulted in CD8^+^ T cell-mediated adaptive immune responses (30). The second one is that OVs facilitate the penetration of proinflammatory cytokines in the TIME, thereby creating a favorable environment for DC activation.

### Recruitment and Activation of T Cells

T cells are highly heterogeneous cell populations with different immunophenotypes and play an indispensable role in adaptive cancer immunity. The main T-cell subsets in the TIME consist of regulatory T cells (Treg), helper T cells (Th), cytotoxic T cells, exhausted T cells, and anergic T cells. These T cell subsets are involved in different immune functions, such as immune effects mediated by cytotoxic T cells or immune suppression mediated by Treg cells (31, 32). T cells can only respond to cancer cells effectually when multiple factors are coordinated. They need to be first primed and activated and then trafficked to for infiltration in the tumors while circumventing the immunosuppressive cells and other inhibitory factors in the TIME (33). However, T cells often dysfunction in cancer (34). OVs that serve as immune-modulating platforms can help overcome barriers and strengthen the T cell-mediated antitumor immunity.

Naïve T cells are primed by T cell receptor (TCR)-mediated recognition of antigenic peptides presented in the context of MHC complex. Antigens of sufficient magnitude play a crucial role in the process of T cell priming. OVs can function as *in situ* vaccines, and, in the appropriate setting, tumors directly serve as an *in situ* source of neoantigen vaccination. Indeed, local OV infection causes direct lysis of tumor cells together with the release of TAAs. For example, an oncolytic adenovirus has been shown to broaden the cancer-specific neoantigen repertoires and enhance the cytotoxic T cell responses to cancer in a murine model of liver cancer (35). In addition, arming OVs with a tumor antigen can further potentiate the T cell immunity. The heterologous prime-boost regimen involving different OVs encoding for the same TAA can help direct cytotoxic T lymphocytes toward cancer-specific new epitopes and away from the virus antigens. This approach has proven successful. For instance, it has been demonstrated that an enhanced tumor antigen-specific T cell response, following priming with an adenovirus encoding for tumor antigen human dopachrome tautomerase (hDCT), in combination with the utilization of a Maraba virus encoding for the same antigen, acted as a booster dose (36).

Differentiated effector T cells possess the ability for circulation and trafficking. Following recruitment by chemokines, the effector T lymphocytes migrate to the established tumor site in order to induce tumor cell killing. OVs can increase infiltration of the T cells in a variety of ways. Local viral infection causes type I IFN response followed by release of T cell-recruiting cytokines and chemokines in the tumor microenvironment. It is well-established that, after oncolytic virotherapy, the immunophenotype of the tumor can switch from the so-called “cold” state to the “hot” state, thereby allowing cytotoxic T cells to infiltrate in the TIME and perform tumor cell killing (37). More importantly, OVs possess the capacity to enhance T cell activation. Indeed, in a past study, the authors showed that although rotavirus vaccines did not significantly increase the overall frequency of T cells in the TIME, they resulted in a highly significant proportion of T cells with increased expression of the activation markers OX40 and CD137 (38).OVs stimulate the secretion of inflammatory mediators such as interleukin-1β, tumor necrosis factor (TNF), and complement components, causing an increase in the expression of selectin on endothelial cells, which acts as the key signal for T cell infiltration. After infiltrating in the TIME, T cells continue to face interference from immunosuppressive cells and other inhibitory factors. OVs can induce the conversion of immunosuppressive cells to proinflammatory phenotypes and promote the development of T cell responses both *in vitro* and *in vivo* (23) Furthermore, exploiting engineered OVs to mediate the direct engagement between T cells and tumor cells, such as OVs armed with a bispecific T cell engager (BiTE), a bispecific affinity reagent that binds to CD3 (or other T cell activators) and target antigens on cancer cells is also a potential strategy. Freedman and colleagues demonstrated that an oncolytic adenovirus expressing EpCAM targeting BiTE led to the activation of both CD4- and CD8-positive T cells to destroy tumor cells (39). Moreover, OVs can be used in conjunction with chimeric antigen receptor-modified T cell therapy (CAR-T). Wing and colleagues have explored the combination of CAR T cells targeting folate receptor alpha (FR-α) with oncolytic adenovirus expressing BiTE targeting EGFR. In the study, they demonstrated that oncolytic adenoviruses carrying BiTE could enhance the CAR T cell activation and proliferation and thereby promoted the re-targeting of CAR-T cells in the absence of FR-α as well as improving the tumor killing by CAR T cells (40).

### Recruitment and Activation of Natural Killer Cells (NKs)

NK cells, which are one of the key components of the innate immune system, are the first line of defense against cancer and other heterologous pathogenic infection (41). Unlike for T cells, the activation of NK cells did not require TCRs, rather it relied on the balance among the activating, co-stimulatory and inhibitory receptors (41). NK cells possess a powerful cytolytic activity. Activated NK cells exert neoplasm killing in a variety of ways, including *via* induction of apoptosis (42) and *via* direct cytolysis by the release of perforin and granzymes (43), Moreover, previous findings have shown that NK cells exhibit intrinsic memory-like properties and possess the ability to undergo rapid clonal expansion in response to a re-challenge (44).

Some preclinical and clinical studies have demonstrated the existence of a cross-talk between OVs and NK cells in cancer immunotherapy. On one hand, NK cells are important immune effectors in the context of oncolytic virotherapy. For instance, a study involving oncolytic NDV combined with immune checkpoint inhibitors demonstrated that the depletion of NK cells could significantly limit their therapeutic effects, which in turn suggests that NK cells are necessary for the regimen including OVs (45). On the other hand, OVs can enhance the proliferation and activity of NK cells. Oncolytic reovirus can promote the anti-tumor activity of NK cells by activating DC *in vitro* (46). Another study conducted in immune-competent mouse models of melanoma found that oncolytic VSV induced the secretion of IL-28 within the tumor microenvironment, which resulted in the promotion of NK cell activation *in vivo* and sensitized tumors to the NK cell-recognition and killing (47). OVs stimulate NK cell-mediated immune responses *via* pattern recognition receptors (PRRs), a class of molecules that are expressed by innate immune cells responsible for sensing heterologous substances. For example, surface toll-like receptor 2 (TLR2), one of the PRRs, mediate NK cell responses stimulated by oncolytic HSV (48). In addition to remodeling the natural occurring NK cells within the TIME, OVs can also promote the homing of NK cells to the tumor site. This homing property correlates with that of the NK cell-recruiting cytokines. For example, it has been demonstrated that oncolytic parvovirus can facilitate the recruitment of NK cells by expressing cytokines IL-2 and MCP-3/CCL7 in a pancreatic ductal adenocarcinoma model (49). Similarly, a combination of the oncolytic adenovirus encoding IL-12 and TRAIL, respectively, was also demonstrated to play an important role in increasing the infiltration of NK cells into the tumors (50). Moreover, OVs possess the ability to potentiate NK cells’ adoptive transfer therapy (51).

### Modulation of Tumor-Associated Macrophages (TAMs)

TAMs represent the crucial elements involved in tumorigenesis that are characterized by the inhibition of antitumor immunity and the promotion of tumor progress (52). The enrichment of TAMs in the tumor site is linked to the poor prognosis and the short survival time in most tumor types (53–55). TAMs produce high amount of immunosuppressive and proangiogenic factors such as IL-10, arginase, transforming growth factor β (TGFβ), or vascular endothelial growth factors (VEGFs); factors such as these enable the tumor-promoting functions of TAMs (56–58). Macrophages exhibit distinct polarization status in response to different stress factors. It is commonly considered that M1 macrophages are proinflammatory and cytotoxic, whereas M2 macrophages are immunosuppressive. Therefore, agents that can induce polarization of TAMs toward M1 type together with the expression of proinflammatory cytokines might be conducive to cancer therapy.

OVs serve as powerful immunological stimuli and are beneficial to shift the phenotype functions of macrophages, as demonstrated by oncolytic paramyxovirus infection of macrophages (59). In this study, virus-treated co-culture conditions induced an anti-tumor phenotype in macrophage *in vitro* accompanied by a higher level of immunostimulatory surface markers and cytokines (59). The mechanisms of how OV infection induce the phenotypic changes of macrophages remain uncertain, but it may be related to virus-induced cytokine release. Indeed, Brown and colleagues investigated the effect of recombinant poliovirus (PVSRIPO) on macrophage activity *in vitro* and found that PVSRIPO infection activated immunosuppressed macrophages in a type I IFN-dominant fashion (23). This OV-mediated remodeling effect is analogously pronounced in solid tumor, wherein OVs create an inflamed milieu that promotes the recruitment and activation of macrophages. For example, it has been reported that the treatment with oncolytic vaccinia virus GLV-1h68 elicited a significant upregulation of proinflammatory cytokines such as IL-3, IL- 6, IFN-γ, and CXCL10, as well as enhancing the infiltration of proinflammatory macrophages to the tumor site in a xenograft colorectal cancer model (60). Similarly, a triple combination therapy including oncolytic HSV increased macrophage infiltration and M1-like polarization, which contributed to glioblastoma eradication (61). Notably, in some cases, OVs may not significantly reduce the amounts of TAMs within the tumor site, instead they remodel TAMs mainly by converting the status of immunosuppressed polarization.

### Modulation of Myeloid-Derived Suppressor Cells (MDSCs)

Myeloid cells are a highly heterologous cell population and can differentiate into MDSCs in response to pathologically persistent stimulation such as chronic infection or inflammation associated with the disease (62). MDSCs are mainly enriched in tumor tissues and other pathological sites and not present in the healthy tissues. Accumulating evidence supports that MDSCs in cancer have emerged as the key contributors to tumor growth and metastasis (63). MDSCs possess the properties of immune suppression. They suppress important immunological processes, particularly in T cell-mediated antitumor responses, by the production of inhibitory factors such as TGFβ, indoleamine 2,3 dioxygenase (IDO), and COX2 (64–66). In addition to intrinsic immune-inhibitory characteristics, MDSCs promote tumor angiogenesis *via* the secretion of diverse growth factors (67). It is now clear that MDSCs reduce the efficacy of cancer immunotherapy; therefore, therapeutics tailored for these cells represent potential therapeutic opportunities.

Previous reports have determined prostaglandin E2 (PGE2) as a critical mediator for MDSCs infiltration by the CXCL-12-CXCR4 pathway (68). In this regard, targeting of PGE2 can help overcome immunosuppression associated with MDSCs, as in the cases of the expression of PGE2 inactivating enzyme 15-hydroxyprostaglandin dehydrogenase (HPGD) by oncolytic vaccinia virus (69). In this study, the engineered OV selectively depleted the MDSCs in the tumor, and, at the same time, the reduction of MDSC populations increased the sensitivity of resistant tumor to oncolytic virotherapy. In addition to serving as a vector for delivery of therapeutic genes specific for MDSCs, OVs per se also possess the capacity for remodeling the frequency and activity of MDSCs. For example, a CpG-rich oncolytic adenovirus can reduce the inhibition of MDSCs by enhancing the TLR9 stimulation in a syngeneic mouse model of melanoma (70). The modulation of MDSCs by OVs may correlate with PRRs in MDSCs. Apart from the abovementioned TLR9 receptor, other OVs limit MDSCs by acting on different PRRs and the relevant signaling molecules, such as in the inhibition of MDSCs by oncolytic reovirus in a TLR3-dependent manner (71) or by oncolytic VSV in a MyD88 signaling-dependent manner (72). MDSCs have intrinsic tumor tropism, which is advantageous, allowing their exploitation as a vehicle for tumor-specific OVs. Eisenstein and colleagues have generated a recombinant oncolytic VSV loaded into MDSCs and found that the MDSCs provided a protective role for the systemic delivery of OVs (73). Importantly, the OVs induced the phenotype of MDSCs switch from the protumor M2 type to antitumor M1 type as a result of virus-mediated inflammatory response; thus, the combination increased the tumor killing as well as the therapeutic index (73).

### Modulation of Cytokines and Immune Checkpoint Molecules

Tumorigenesis is often aided by the shaping of the TIME by cytokines. Several reports have demonstrated that the oncogene-driven expression of cytokines and/or chemokines is associated with an increase in the number of immunosuppressive cells, such as Gr-1^+^CD11b^+^ myeloid cells (74), as well as a decrease in the frequency of immune effector cells such as CD103^+^ DCs (75). Multifunctional cytokines in the tumor microenvironment are not only involved in tumor progress, but they also act as pivotal mediators for the antitumor responses. Oncolytic virotherapy induce immunogenic cell death (ICD) of cancerous cells together with abound release of danger-associated molecular patterns (DAMPs), such as ATP, nuclear high mobility group box 1 (HMBG1), and calreticulin (76–78). These proinflammatory substances exhibit the intrinsic properties of immune-stimulating, which is beneficial to the recruitment and activation of immune effector cells to the tumor site. Moreover, viral particles are detected by PRRs on the surface and/or cytoplasm of innate immune cells, which culminates into intracellular immune responses against viruses in a type I IFN-dominant fashion (79). This innate antiviral machinery induces the generation of an inflamed TIME by driving the expression of IFN-inducible immunomodulatory cytokines (26). In addition, OVs can serve as a platform for the expression of various cytokines. For example, the first FDA-approved OV agent derived from oncolytic HSV-1 was genetically modified to encode for GM-CSF (80). However, it remains to be understood how OV-induced production of various cytokines by different cell compositions can be coordinated to determine the immune landscapes of the TIME.

Tumor cells can evade immune surveillance by virtue of immune checkpoint molecules that link to ligands expressed on the immune cells. The typical checkpoint molecules consist of programmed cell death protein 1 (PD-1) and its ligand (PD-L1), cytotoxic T lymphocyte- associated protein 4 (CTLA-4), and lymphocyte activation gene 3 (LAG3). In fact, the immune checkpoint blockade can reverse the immune effector cell anergy by targeting the inhibitory signaling pathways and have been investigated with success in multiple tumor types (81–83). However, tumors with low mutation burden often correlates with the resistance of immune checkpoint blockade due to the dearth of sufficient antigen recognition by T lymphocytes. Oncolytic virotherapy can increase the expression of checkpoint molecules within the tumor site. For example, oncolytic Maraba virus induce the upregulation of PD-L1 in ovarian cancers (84) and oncolytic NDV induce the upregulation of CTLA-4 in melanoma (45). A past study demonstrated OV-mediated production of type I IFN as a key mediator for the upregulation of immune checkpoint molecules (85). Moreover, following OV infection, adaptive immune resistance is also involved in the increase of checkpoint molecules, which may correlate with the compensatory immunosuppressive pathways (86).

## Conclusions

Accumulating evidence support the potential of OVs as a promising therapeutic agent. The potent antitumor activity of OVs can be attributed to their unique mechanisms of action, including direct oncolysis and the induction of immune responses. OVs remodel the immune landscape of the TIME by regulating immune cells and the relevant cytokines within the tumor microenvironment. These therapeutic viruses possess the ability to recruit and activate immune effector cells, particularly CD8^+^ T lymphocytes. Simultaneously, they reduce the amounts of immunosuppressive cells and alternatively induce the phenotype of the cells that shift from the protumor status to the antitumor status. OV-induced increased infiltration of immune effector cells is often accompanied by abound secretion of proinflammatory cytokines. It is now evident that oncolytic virotherapy can create an inflamed TIME, or a “hot” tumor, which is expected to yield a superior efficacy in combination with cancer immunotherapy. Further development of oncology and virotherapy is expected to provide a deeper understanding of the interaction between OVs and the TIME in the future.

## Author Contributions

XLW retrieved the relevant paper and finished the original draft. BZ modified the original draft and added substantial contents. PC provided constructive guidance and critical advice on the framework this review. All authors contributed to the article and approved the submitted version.

## Funding

This work was supported by the National Science and Technology Major Projects of New Drugs (2018ZX09201018-013), the National Science and Technology Major Project for Infectious Diseases Control (2017ZX10203206-004) and the National Natural Science Foundation of China (81101728).

## Conflict of Interest

The authors declare that the research was conducted in the absence of any commercial or financial relationships that could be construed as a potential conflict of interest.
